# Supplementary material for: Trends in Incidence and Case Fatality Rates of Heart Disease and Its Subtypes in Korea, 2006–2015
Source: Int J Environ Res Public Health. 2020 Nov 15;17(22):8451. doi: 10.3390/ijerph17228451 (PMC7696411; doi:10.3390/ijerph17228451)
Supplement: Supplementary file 1 [file ijerph-17-08451-s001.pdf]

Supplementary Material

Table S1. The numbers of Korean population by sex and age-groups.

Variables	2006	2007	2008	2009	2010	2011	2012	2013	2014	2015
Total										
Populations	49,238,227	49,672,388	50,001,057	50,290,771	50,581,191	50,908,645	51,169,141	51,448,491	51,757,146	52,034,424
Age groups										
0–39	28,653,292	28,339,746	27,909,053	27,455,541	27,025,679	26,592,749	26,180,188	25,769,610	25,402,024	25,088,060
40–49	8,497,204	8,602,222	8,751,947	8,823,843	8,814,941	8,851,311	8,852,699	8,954,630	8,990,011	8,932,912
50–59	5,573,511	5,889,864	6,198,789	6,575,316	7,028,311	7,508,879	7,788,986	8,041,766	8,263,032	8,392,795
60–69	3,733,515	3,872,908	3,987,188	4,072,221	4,166,825	4,197,449	4,314,938	4,466,479	4,712,416	5,089,893
70–79	2,072,709	2,208,766	2,338,886	2,484,188	2,603,758	2,755,942	2,954,146	3,056,411	3,129,667	3,159,341
80+	707,996	758,882	815,194	879,662	941,677	1,002,315	1,078,184	1,159,595	1,259,996	1,371,423
Male										
Populations	24,732,975	24,944,131	25,102,682	25,241,212	25,379,265	25,536,889	25,647,133	25,780,143	25,934,973	26,065,615
Age groups										
0–39	14,855,569	14,701,381	14,483,588	14,252,983	14,032,657	13,809,937	13,587,974	13,385,857	13,204,355	13,048,601
40–49	4,345,054	4,386,375	4,457,134	4,494,809	4,495,325	4,518,432	4,513,485	4,554,512	4,572,951	4,543,353
50–59	2,789,750	2,955,447	3,114,533	3,302,578	3,526,284	3,766,122	3,913,084	4,045,834	4,156,131	4,222,870
60–69	1,740,418	1,817,100	1,883,552	1,935,652	1,990,985	2,014,273	2,080,635	2,160,740	2,290,733	2,473,830
70–79	795,206	863,706	928,513	1,000,766	1,063,285	1,139,290	1,237,935	1,290,711	1,331,895	1,356,673
80+	206,978	220,122	235,362	254,424	270,729	288,835	314,020	342,489	378,908	420,288
Female										
Populations	24,505,252	24,728,257	24,898,375	25,049,559	25,201,926	25,371,756	25,522,008	25,668,348	25,822,173	25,968,809
Age groups										
0–39	13,797,723	13,638,365	13,425,465	13,202,558	12,993,022	12,782,812	1,592,214	12,383,753	12,197,669	12,039,459
40–49	4,152,150	4,215,847	4,294,813	4,329,034	4,319,616	4,332,879	4,339,214	4,400,118	4,417,060	4,389,559
50–59	2,783,761	2,934,417	3,084,256	3,272,738	3,502,027	3,742,757	3,875,902	3,995,932	4,106,901	4,169,925
60–69	1,993,097	2,055,808	2,103,636	2,136,569	2,175,840	2,183,176	2,234,303	2,305,739	2,421,683	2,616,063
70–79	1,277,503	1,345,060	1,410,373	1,483,422	1,540,473	1,616,652	1,716,211	1,765,700	1,797,772	1,802,668
80+	501,018	538,760	579,832	625,238	670,948	713,480	764,164	817,106	881,088	951,135

Table S2. Annual incident cases and incidence rates of Ischemic Heart Disease (I20-I25).

**Variables**	**2006**	**2007**	**2008**	**2009**	**2010**	**2011**	**2012**	**2013**	**2014**	**2015**
Total Incident cases										
Total, number	80,040	82,565	80,597	81,182	83,199	84,165	86,280	83,150	86,025	82,296
Mean age, year	62.8	63.2	63.5	63.7	63.8	64.1	64.2	64.6	64.6	64.9
Age group, %										
0–39	3.0	2.9	2.7	2.5	2.5	2.1	2.1	2.2	2.1	1.9
40–49	11.7	11.3	10.9	10.5	10.3	9.4	9.4	9.6	9.3	8.8
50–59	23.4	22.8	22.5	23.2	23.3	24.0	24.0	24.2	24.1	23.6
60–69	30.9	30.4	30.3	29.4	29.2	28.1	28.1	27.3	27.6	28.0
70–79	23.3	24.3	25.0	25.2	25.3	26.1	26.1	26.1	25.6	25.3
80+	7.8	8.3	8.6	9.1	9.4	10.2	10.2	10.8	11.4	12.3
Total Incidence rate (per 100,000 persons-year)										
Crude rate	162.6	166.2	161.2	161.4	164.5	165.3	168.6	161.6	166.2	158.2
Age-standardized rate	143.9	141.9	132.9	128.3	126.5	123.1	121.1	112.8	112.5	103.5
Age group, year										
0–39	8.3	8.4	7.9	7.4	7.8	7.3	7.1	7.0	7.0	6.3
40–49	110.2	108.4	100.4	96.3	96.8	89.7	91.9	88.8	88.6	81.4
50–59	335.6	320.0	292.3	286.8	275.5	273.8	265.6	249.8	250.5	231.7
60–69	661.8	648.8	612.5	586.8	583.3	565.6	562.9	507.4	503.8	452.5
70–79	901.3	908.5	860.0	824.4	808.6	786.1	761.1	709.0	703.5	659.7
80+	878.1	898.3	851.9	842.8	833.1	830.8	819.1	774.1	779.5	737.8
Men Incident cases										
Men, number	44,342	45,658	45,288	46,744	48,569	49,585	51,394	50,220	52,388	51,118
Men, %	55.4	55.3	56.2	57.6	58.4	58.9	59.6	60.4	60.9	62.1
Mean age, year	60.3	60.6	60.8	61.0	61.3	61.5	61.8	61.7	61.9	62.3
Men Incidence rate (per 100,000 person-year)										
Crude rate	179.3	183.0	180.4	185.2	191.4	194.2	200.4	194.8	202.0	196.1
Age-standardized rate	176.1	173.2	164.7	162.6	162.4	159.3	158.5	149.2	149.7	140.7
Women Incident cases										
Women, number	35,698	36,907	35,309	34,438	34,630	34,580	34,886	32,930	33,637	31,178

Women, %	44.6	44.7	43.8	42.4	41.6	41.1	40.4	39.6	39.1	37.9
Mean age, year	66.1	66.5	67.0	67.3	67.4	67.8	67.9	68.4	68.6	69.2
Women Incidence rate (per 100,000 person-year)										
Crude rate	145.7	149.3	141.8	137.5	137.4	136.3	136.7	128.3	130.3	120.1
Age-standardized rate	115.1	113.6	104.1	97.2	94.1	90.2	87.2	79.0	77.9	69.1

Table S3. Annual incident cases and incidence rates of Heart Failure (I50).

Variables	2006	2007	2008	2009	2010	2011	2012	2013	2014	2015
Total Incident cases of HF										
Total, number	13,351	14,023	14,637	14,459	15,080	16,060	16,394	16,432	17,072	18,123
Mean age, year	73.3	73.8	74.0	74.5	74.5	74.9	74.9	75.3	75.4	76.1
Age group, %										
0–39	1.6	1.9	1.8	1.6	1.7	1.6	1.8	1.6	1.6	1.5
40–49	3.6	3.5	3.4	3.1	3.4	3.2	3.3	3.0	3.1	2.7
50–59	7.5	6.8	6.8	6.9	6.8	7.2	7.0	6.9	6.7	6.6
60–69	16.6	16.2	15.5	14.6	14.7	13.5	12.7	12.1	11.6	12.1
70–79	37.0	35.0	35.5	34.5	33.8	33.2	33.6	33.2	32.1	30.8
80+	33.6	36.5	36.9	39.2	39.7	41.3	41.6	43.1	44.9	46.4
Total Incidence rate (per 100,000 persons-year)										
Crude rate	27.1	28.2	29.3	28.8	29.8	31.5	32.0	31.9	33.0	34.8
Age-standardized rate	25.6	25.5	25.2	23.6	23.4	23.7	22.9	21.8	21.5	21.7
Age group, year										
0–39	0.8	0.9	0.9	0.9	0.9	1.0	1.2	1.1	1.1	1.1
40–49	5.7	5.8	5.7	5.1	5.9	5.7	6.1	5.5	5.8	5.5
50–59	18.0	16.2	16.1	15.1	14.6	15.3	14.8	14.2	13.9	14.3
60–69	59.3	58.7	57.1	51.8	53.1	51.8	48.1	44.6	42.0	43.1
70–79	238.4	222.1	221.9	201.0	195.5	193.4	186.5	178.7	175.1	176.5
80+	633.2	675.3	663.0	645.0	635.4	661.9	632.6	610.2	608.7	612.6
Male Incident cases										
Male, number	4,471	4,648	5,158	5,033	5,545	5,976	6,201	6,343	6,487	6,929
Mean age, year	69.2	69.6	70.0	70.3	70.3	70.5	70.6	71.1	71.4	71.8
Male, %	33.5	33.1	35.2	34.8	36.8	37.2	37.8	38.6	38.0	38.2
Male Incidence rate (per 100,000 person-year)										
Crude rate	18.1	18.6	20.5	19.9	21.8	23.4	24.2	24.6	25.0	26.6
Age-standardized rate	21.8	21.7	22.7	21.1	22.0	22.6	22.1	21.5	20.9	21.1
Female Incident cases										
Female, number	8,880	9,375	9,479	9,426	9,535	10,084	10,193	10,089	10,585	11,194
Mean age, year	75.4	75.9	76.2	76.8	77.0	77.4	77.5	78.0	78.4	78.7
Female, %	66.5	66.9	64.8	65.2	63.2	62.8	62.2	61.4	62.0	61.8
Female Incidence rate (per 100,000 person-year)										
Crude rate	36.2	37.9	38.1	37.6	37.8	39.7	39.9	39.3	41.0	43.1
Age-standardized rate	27.4	27.4	26.3	24.6	23.7	23.8	22.8	21.4	21.3	21.4

Table S4. Annual incident cases and incidence rates of HHD (I11, I13).

Variables	2006	2007	2008	2009	2010	2011	2012	2013	2014	2015
Total Incident cases of HHD										
Total, number	8,350	8,544	7,996	7,142	6,800	7,115	6,979	6,347	6,161	6,317
Mean age, year	72.1	73.3	73.7	73.6	74.0	73.7	73.6	73.2	73.0	74.1
Age group, %										
0–39	1.4	1.2	1.3	1.5	1.4	1.7	2.0	2.2	2.3	2.4
40–49	4.8	4.6	4.4	4.6	4.2	4.6	4.4	4.8	4.2	4.3
50–59	10.4	9.0	8.8	9.2	8.6	9.8	10.4	10.6	10.0	9.5
60–69	17.4	15.8	14.3	14.5	13.6	14.2	13.6	13.8	13.6	13.6
70–79	35.7	34.7	33.5	32.7	33.6	30.1	30.0	29.5	27.2	28.4
80+	30.3	34.8	37.6	37.6	38.6	39.6	39.7	39.0	42.7	41.8
Total Incidence rate (per 100,000 persons-year)										

Crude rate	17.0	17.2	16.0	14.2	13.4	14.0	13.6	12.3	11.9	12.1
Age-standardized rate	15.9	15.4	13.7	11.6	10.5	10.5	9.8	8.5	7.9	7.7
Age group, year										
0–39	0.4	0.4	0.4	0.4	0.4	0.5	0.5	0.6	0.6	0.6
40–49	4.7	4.5	4.0	3.7	3.2	3.7	3.4	3.4	2.9	3.1
50–59	15.6	13.0	11.4	10.0	8.3	9.3	9.3	8.4	7.4	7.2
60–69	38.9	34.9	28.7	25.4	22.2	24.0	22.0	19.7	17.8	16.9
70–79	143.7	134.1	114.5	94.1	87.6	77.8	70.8	61.2	53.6	56.7
80+	357.6	391.4	368.9	305.2	278.8	281.3	256.7	213.7	208.6	192.4
Male Incident cases										
Male, number	2,759	2,726	2,552	2,239	2,243	2,360	2,213	2,136	1,995	2,203
Mean age, year	66.9	68.0	68.3	67.8	68.6	67.9	67.5	67.5	67.7	68.6
Male, %	33.0	31.9	31.9	31.3	33.0	33.2	31.7	33.7	32.4	34.9
Male Incidence rate (per 100,000 person-year)										
Crude rate	11.2	10.9	10.2	8.9	8.8	9.2	8.6	8.3	7.7	8.5
Age-standardized rate	12.7	12.3	11.0	9.0	8.7	8.8	7.8	7.1	6.4	6.7
Female Incident cases										
Female, number	5,591	5,818	5,444	4,903	4,557	4,755	4,766	4,211	4,166	4,114
Mean age, year	74.7	75.7	76.3	76.2	76.7	76.6	76.5	76.1	77.0	77.0
Female, %	67.0	68.1	68.1	68.7	67.0	66.8	68.3	66.3	67.6	65.1
Female Incidence rate (per 100,000 person-year)										
Crude rate	22.8	23.5	21.9	19.6	18.1	18.7	18.7	16.4	16.1	15.8
Age-standardized rate	17.3	17.0	15.0	12.8	11.3	11.3	10.7	9.1	8.6	8.1

Table S5. Annual incident cases and incidence rates of Arrhythmia (I47~I49).

Variables	2006	2007	2008	2009	2010	2011	2012	2013	2014	2015
Total Incident cases										
Total, number	12,156	13,476	14,177	15,607	16,485	18,258	19,747	20,424	21,755	22,325
Mean age, year	59.7	60.6	61.2	61.1	61.1	61.8	62.4	62.6	63.2	63.9
Age group, %										
0–39	13.5	12.8	12.3	12.4	11.9	11.3	10.7	10.6	9.8	9.0
40–49	12.5	11.7	11.4	11.4	11.1	10.5	10.0	9.5	9.5	8.8
50–59	16.8	16.8	16.2	16.8	18.5	18.3	18.3	18.6	17.8	17.3
60–69	23.0	21.8	21.6	21.3	20.9	20.3	19.9	19.8	19.8	21.3
70–79	23.0	24.1	24.9	24.0	23.8	24.6	25.5	25.2	25.8	25.1
80+	11.2	12.7	13.6	14.0	13.7	15.1	15.6	16.3	17.3	18.6
Total Incidence rate (per 100,000 persons-year)										
Crude rate	24.7	27.1	28.4	31.0	32.6	35.9	38.6	39.7	42.0	42.9
Age-standardized rate	22.6	24.7	24.5	26.0	26.5	28.4	29.5	29.6	30.4	30.1
Age group, year										
0–39	5.7	6.1	6.3	7.1	7.3	7.7	8.1	8.4	8.4	8.0
40–49	17.9	18.3	18.5	20.1	20.7	21.6	22.2	21.7	22.9	22.0
50–59	36.7	38.5	37.0	39.9	43.5	44.5	46.5	47.3	47.0	46.0
60–69	74.8	76.0	76.7	81.7	82.7	88.3	90.9	90.5	91.4	93.4
70–79	134.9	147.2	150.9	151.0	150.8	162.8	170.8	168.6	179.6	177.1
80+	192.8	226.4	237.2	248.5	240.2	274.4	285.3	286.7	298.1	302.5
Male Incident cases										
Men, number	5,943	6,442	6,633	7,584	8,189	9,082	9,895	10,283	10,826	11,373
Men, %	48.9	47.8	46.8	48.6	49.7	49.7	50.1	50.3	49.8	50.9
Mean age, year	56.2	57.2	57.9	58.3	58.2	58.9	59.4	59.8	60.2	61.2
Men Incidence rate (per 100,000 person-year)										

Crude rate	24.0	25.8	26.4	30.0	32.3	35.6	38.6	39.9	41.7	43.6
Age-standardized rate	24.7	25.9	25.9	28.5	29.3	31.6	33.0	33.2	33.8	34.1
Female Incident cases										
Women, number	6,213	7,034	7,544	8,023	8,296	9,176	9,852	10,141	10,929	10,952
Women, %	51.1	52.2	53.2	51.4	50.3	50.3	49.9	49.7	50.2	49.1
Mean age, year	63.1	63.7	64.1	63.8	63.9	64.8	65.3	65.4	66.2	66.7
Women Incidence rate (per 100,000 person-year)										
Crude rate	25.4	28.4	30.3	32.0	32.9	36.2	38.6	39.5	42.3	42.2
Age-standardized rate	20.7	22.5	23.2	24.0	24.0	25.5	26.4	26.4	27.3	26.4

Table S6. Annual incident cases and incidence rates of Pulmonary HD (I26~I28).

Variables	2006	2007	2008	2009	2010	2011	2012	2013	2014	2015
Total Incident cases of Pulmonary HD										
Total, number	1,799	2,277	2,605	2,822	3,277	3,746	3,727	3,966	4,253	4,471
Mean age, year	62.8	63.6	63.8	65.3	66.2	65.7	66.6	67.5	68.0	68.3
Age group, %										
0–39	11.8	12.4	11.7	10.9	8.9	10.3	9.4	8.4	8.0	8.3
40–49	10.8	7.5	8.1	6.8	6.6	7.1	6.4	7.0	6.8	5.5
50–59	10.3	10.9	10.4	9.7	10.0	10.4	10.1	9.1	9.5	9.8
60–69	21.2	20.6	20.2	20.3	19.7	17.8	16.7	16.8	16.0	15.8
70–79	30.9	32.6	33.3	33.0	34.6	33.0	34.9	34.5	33.9	32.3
80+	14.9	16.1	16.2	19.4	20.2	21.3	22.5	24.2	25.9	28.2
Total Incidence rate (per 100,000 persons-year)										
Crude rate	3.7	4.6	5.2	5.6	6.5	7.4	7.3	7.7	8.2	8.6
Age-standardized rate	3.4	4.1	4.5	4.7	5.2	5.8	5.5	5.6	5.8	5.9
Age group, year										
0–39	0.7	1.0	1.1	1.1	1.1	1.5	1.3	1.3	1.3	1.5
40–49	2.3	2.0	2.4	2.2	2.4	3.0	2.7	3.1	3.2	2.8
50–59	3.3	4.2	4.4	4.2	4.7	5.2	4.8	4.5	4.9	5.2
60–69	10.2	12.1	13.2	14.1	15.5	15.9	14.4	15.0	14.4	13.9
70–79	26.8	33.6	37.1	37.4	43.5	44.9	44.0	44.7	46.1	45.8
80+	37.9	48.2	51.6	62.3	70.4	79.6	77.8	82.8	87.3	92.0
Male Incident cases										
Male, number	698	869	1,036	1,097	1,237	1,460	1,452	1,507	1,625	1,716
Mean age, year	59.0	59.7	59.4	61.8	62.2	62.0	62.6	63.4	64.2	64.3
Male, %	38.8	38.2	39.8	38.9	37.7	39.0	39.0	38.0	38.2	38.4
Male Incidence rate (per 100,000 person-year)										
Crude rate	2.8	3.5	4.1	4.3	4.9	5.7	5.7	5.8	6.3	6.6
Age-standardized rate	3.1	3.7	4.2	4.4	4.7	5.4	5.1	5.1	5.3	5.4
Female Incident cases										
Female, number	1,101	1,408	1,569	1,725	2,040	2,286	2,275	2,459	2,628	2,755
Mean age, year	65.2	66.0	66.7	67.4	68.7	68.0	69.1	70.1	70.4	70.8
Female, %	61.2	61.8	60.2	61.1	62.3	61.0	61.0	62.0	61.8	61.6
Female Incidence rate (per 100,000 person-year)										
Crude rate	4.5	5.7	6.3	6.9	8.1	9.0	8.9	9.6	10.2	10.6
Age-standardized rate	3.6	4.4	4.7	5.0	5.6	6.2	5.8	6.0	6.1	6.2

Table S7. Annual incident cases and incidence rates of HD others (I00~I09, I30~I33, I39, I40~I46, I51).

Variables	2006	2007	2008	2009	2010	2011	2012	2013	2014	2015
Total Incident cases of Others of HD										
Total, number	10,007	10,653	10,939	11,234	11,938	13,006	13,503	13,609	14,907	15,609
Mean age, year	60.2	61.1	61.5	61.7	62.6	62.6	63.3	63.8	64.4	65.4
Age group, %										
0–39	13.3	13.0	12.6	12.6	11.7	11.3	10.8	10.8	9.6	9.1
40–49	12.5	11.5	10.9	10.7	9.9	10.4	9.2	8.7	8.8	8.2
50–59	16.7	16.1	15.9	15.7	15.5	16.2	16.0	15.8	16.0	15.0
60–69	22.4	21.3	21.3	20.2	20.1	18.7	18.9	17.9	18.0	17.9
70–79	22.9	23.1	24.5	25.3	26.1	25.8	26.5	27.5	26.9	26.8
80+	12.4	15.0	14.8	15.5	16.7	17.5	18.5	19.3	20.6	23.0
Total Incidence rate (per 100,000 persons-year)										
Crude rate	20.3	21.4	21.9	22.3	23.6	25.5	26.4	26.5	28.8	30.0
Age-standardized rate	18.7	19.2	19.0	18.8	19.2	20.3	20.2	19.7	20.7	20.9
Age group, year										
0–39	4.6	4.9	4.9	5.2	5.2	5.5	5.6	5.7	5.7	5.7
40–49	14.7	14.2	13.7	13.6	13.4	15.3	14.1	13.2	14.6	14.3
50–59	29.9	29.1	28.1	26.8	26.4	28.1	27.8	26.7	28.9	27.9
60–69	59.9	58.6	58.5	55.7	57.6	57.8	59.2	54.6	56.9	54.8
70–79	110.5	111.6	114.6	114.4	119.8	121.9	121.0	122.3	128.3	132.3
80+	175.1	211.0	198.1	198.5	211.5	227.0	232.0	227.1	243.3	262.1
Male Incident cases										
Male, number	4,722	4,935	5,171	5,387	5,885	6,477	6,864	6,924	7,655	8,028
Mean age, year	56.6	57.4	57.9	58.2	58.8	59.0	59.8	60.2	61.0	61.6
Male, %	47.2	46.3	47.3	48.0	49.3	49.8	50.8	50.9	51.4	51.4
Male Incidence rate (per 100,000 person-year)										
Crude rate	19.1	19.8	20.6	21.3	23.2	25.4	26.8	26.9	29.5	30.8
Age-standardized rate	19.7	20.1	20.3	20.4	21.6	22.8	23.4	22.8	24.2	24.6
Female Incident cases										
Female, number	5,285	5,718	5,768	5,847	6,053	6,529	6,639	6,685	7,252	7,581
Mean age, year	63.4	64.3	64.7	65.0	66.3	66.3	67.0	67.4	67.9	69.4
Female, %	52.8	53.7	52.7	52.0	50.7	50.2	49.2	49.1	48.6	48.6
Female Incidence rate (per 100,000 person-year)										
Crude rate	21.6	23.1	23.2	23.3	24.0	25.7	26.0	26.0	28.1	29.2
Age-standardized rate	17.6	18.2	17.7	17.3	17.1	17.9	17.4	16.9	17.6	17.4

Table S8. Annual incident cases and incidence rates of Valvular HD (I34~I38).

Variables	2006	2007	2008	2009	2010	2011	2012	2013	2014	2015
Total Incident cases of Valvular HD										
Total, number	3,122	3,187	3,509	3,587	3,597	3,749	3,901	3,918	4,048	4,437
Mean age, year	60.4	61.2	62.0	63.0	63.5	65.0	65.3	65.6	67.4	67.2
Age group, %										
0–39	12.4	12.6	11.3	9.5	9.4	8.0	7.8	8.1	5.4	6.0
40–49	11.6	10.7	10.1	10.3	8.9	8.2	8.1	7.0	7.3	7.1
50–59	17.4	15.6	17.0	16.0	16.0	14.9	14.6	14.6	13.5	13.7
60–69	23.4	22.9	22.7	22.9	22.5	21.4	20.1	19.6	20.1	20.8
70–79	24.1	24.8	24.9	25.8	27.1	29.2	30.0	31.1	31.0	28.7
80+	11.1	13.5	14.0	15.6	16.0	18.3	19.3	19.7	22.6	23.6
Total Incidence rate (per 100,000 persons-year)										
Crude rate	6.3	6.4	7.0	7.1	7.1	7.4	7.6	7.6	7.8	8.5

Age-standardized rate	5.8	5.7	6.0	5.9	5.7	5.7	5.7	5.6	5.4	5.8
Age group, year										
0–39	1.4	1.4	1.4	1.2	1.2	1.1	1.2	1.2	0.9	1.1
40–49	4.2	4.0	4.1	4.2	3.6	3.5	3.6	3.0	3.3	3.5
50–59	9.8	8.4	9.6	8.7	8.2	7.4	7.3	7.1	6.6	7.2
60–69	19.6	18.9	20.0	20.2	19.5	19.1	18.2	17.2	17.3	18.2
70–79	36.2	35.7	37.4	37.2	37.5	39.8	39.6	39.8	40.1	40.4
80+	48.9	56.7	60.5	63.4	61.2	68.3	69.9	66.5	72.5	76.4
Male Incident cases										
Male, number	1,347	1,369	1,603	1,591	1,644	1,672	1,778	1,822	1,850	2,074
Mean age, year	56.6	58.0	58.3	59.2	60.8	61.0	61.2	62.0	63.6	63.4
Male, %	43.1	43.0	45.7	44.4	45.7	44.6	45.6	46.5	45.7	46.7
Male Incidence rate (per 100,000 person-year)										
Crude rate	5.4	5.5	6.4	6.3	6.5	6.5	6.9	7.1	7.1	8.0
Age-standardized rate	5.6	5.5	6.2	5.9	5.9	5.8	6.0	5.9	5.7	6.2
Female Incident cases										
Female, number	1,775	1,818	1,906	1,996	1,953	2,077	2,123	2,096	2,198	2,363
Mean age, year	63.4	63.7	65.1	66.0	65.8	68.2	68.7	68.7	70.5	70.5
Female, %	56.9	57.0	54.3	55.6	54.3	55.4	54.4	53.5	54.3	53.3
Female Incidence rate (per 100,000 person-year)										
Crude rate	7.2	7.4	7.7	8.0	7.7	8.2	8.3	8.2	8.5	9.1
Age-standardized rate	5.9	5.8	5.8	5.8	5.5	5.5	5.4	5.2	5.1	5.3

Table S9. Trends of case-fatality rates by subtypes of HD in Korea, 2006 to 2015.

Variables	2006	2007	2008	2009	2010	2011	2012	2013	2014	2015
Heart Disease (I00-I09, I11, I13, I20~I51)										
7–day	1.2	1.3	1.3	1.3	1.3	1.3	1.3	1.3	1.3	1.4
30–day	2.2	2.4	2.3	2.2	2.3	2.3	2.3	2.2	2.3	2.5
1–year	4.3	4.4	4.0	3.8	3.8	4.0	3.7	3.7	3.7	3.9
3–year	6.5	6.4	5.9	5.6	5.6	5.6	5.2	5.3	5.1	–
5–year	8.1	7.9	7.3	6.9	6.8	6.9	6.5	-	-	-
Heart Failure (I50)										
7-day	1.8	2.7	3.0	3.2	3.1	3.2	3.4	3.2	3.4	3.3
30-day	3.9	6.1	6.2	6.5	6.4	6.8	6.9	6.6	6.8	6.7
1-year	11.1	13.9	13.2	13.1	13.0	13.2	12.8	12.7	213.4	13.0
3-year	17.7	20.1	19.4	19.2	18.9	19.0	18.5	18.7	19.1	-
5-year	21.8	24.5	23.6	23.3	22.7	22.8	22.3	-	-	-
Heart Disease Others (I00-I09, I30-I33, I39, I40-I46, I51)										
7-day	1.2	2.0	2.3	2.7	3.0	3.3	3.8	3.6	3.7	4.0
30-day	2.5	4.0	4.4	4.6	5.3	5.3	6.1	5.8	6.1	6.4
1-year	5.7	8.1	8.2	8.0	8.7	9.2	9.1	9.1	9.2	9.5
3-year	9.1	11.2	11.5	11.1	12.0	12.2	11.6	12.1	11.8	-
5-year	11.3	13.4	13.8	13.0	14.0	14.2	13.6	-	-	-
Valvular Heart Disease (I34-I38)										
7-day	0.8	1.2	1.0	1.0	1.5	1.4	1.7	1.7	1.6	2.0
30-day	1.9	3.0	2.8	2.9	3.2	3.6	4.1	3.8	4.0	4.1
1-year	4.9	6.9	6.2	6.2	7.0	7.4	8.1	6.7	7.8	7.7
3-year	7.9	9.7	9.8	9.5	10.1	10.5	11.7	10.2	10.9	-
5-year	9.8	12.3	12.1	11.6	12.5	13.2	14.3	-	-	-
Hypertensive Heart Disease (I11, I13)										

7-day	0.8	1.2	1.0	1.2	1.3	1.2	1.1	1.0	1.1	1.1
30-day	2.2	3.3	3.2	3.2	3.3	3.0	2.8	2.6	2.6	3.1
1-year	7.2	8.8	8.0	8.2	8.1	8.1	6.7	6.2	6.6	6.4
3-year	12.0	12.8	12.8	12.9	12.8	11.7	10.3	9.7	9.5	-
5-year	14.5	15.3	15.4	15.8	15.9	14.6	12.4	-	-	-
Arrhythmia (I47-I49)										
7-day	0.4	0.6	0.7	0.6	0.7	0.6	0.6	0.7	0.6	0.7
30-day	0.9	1.3	1.2	1.2	1.2	1.3	1.2	1.3	1.1	1.3
1-year	2.5	3.3	3.3	3.1	3.0	3.1	3.0	3.0	2.9	3.1
3-year	4.6	5.6	5.2	5.3	5.2	5.1	4.9	5.1	5.0	-
5-year	6.4	7.3	7.0	7.1	6.7	6.7	6.5	-	-	-
Pulmonary Heart Disease (I26~I18)										
7-day	1.6	1.7	2.0	1.5	2.0	2.1	2.6	1.9	1.8	1.4
30-day	2.6	3.2	3.2	3.0	3.6	3.5	3.4	3.3	3.2	2.8
1-year	5.0	6.3	5.6	5.9	6.6	6.2	6.1	6.2	5.2	4.9
3-year	7.3	9.2	7.9	8.4	9.1	8.8	8.0	8.4	7.2	-
5-year	8.8	11.7	9.7	9.7	10.9	10.5	9.5	-	-	-
Ischemic Heart Disease (I20~I25)										
7-day	1.0	1.2	1.1	1.0	1.0	1.0	1.0	1.0	0.9	1.0
30-day	1.8	2.1	1.9	1.8	1.7	1.7	1.7	1.6	1.6	1.7
1-year	3.3	3.7	3.3	3.0	2.9	2.9	2.7	2.7	2.6	2.8
3-year	4.9	5.2	4.7	4.4	4.2	4.1	3.9	3.8	3.7	-
5-year	6.2	6.4	5.9	5.4	5.1	5.1	4.9	-	-	-

Table S10. Trends of case-fatality rates by subtypes of HD in Korean male, 2006 to 2015.

Variables	2006	2007	2008	2009	2010	2011	2012	2013	2014	2015
Heart Disease (I00-I09, I11, I13, I20~I51)										
7-day	1.0	1.1	1.0	0.9	1.0	1.0	1.1	1.0	1.0	1.1
30-day	1.8	2.0	1.9	1.7	1.8	1.8	1.8	1.8	1.8	1.9
1-year	3.4	3.5	3.3	2.9	3.0	3.2	3.0	2.9	2.9	3.0
3-year	5.3	5.2	4.8	4.3	4.4	4.4	4.2	4.2	4.0	-
5-year	6.6	6.4	6.0	5.4	5.4	5.5	5.1	-	-	-
Heart Failure (I50)										
7-day	1.8	2.8	2.7	2.7	3.0	3.0	3.1	3.1	3.3	2.9
30-day	3.5	6.2	6.0	6.1	5.7	6.2	6.3	6.1	6.4	6.1
1-year	10.2	13.5	12.8	12.1	12.0	12.3	12.1	12.1	13.0	11.9
3-year	16.9	19.1	19.0	18.1	17.4	17.7	17.4	17.7	18.3	-
5-year	21.0	23.1	23.2	22.2	21.1	21.5	20.9	-	-	-
Heart Disease Others (I00-I09, I30-I33, I39, I40-I46, I51)										
7-day	1.3	2.0	2.8	2.5	3.2	3.5	3.7	3.8	4.2	4.0
30-day	2.8	3.7	4.7	4.6	5.4	5.8	5.8	6.3	6.7	6.4
1-year	5.5	7.6	8.7	7.9	8.8	9.4	9.0	9.4	9.6	9.2
3-year	8.7	10.7	11.9	10.7	11.7	12.1	11.5	12.2	11.7	-
5-year	10.8	12.8	13.9	12.5	13.5	14.0	13.2	-	-	-
Valvular Heart Disease (I34-I38)										
7-day	0.6	1.2	0.9	1.0	1.5	1.1	1.5	1.1	1.2	1.7
30-day	1.8	2.8	2.2	2.9	3.1	3.0	3.8	3.0	3.0	3.8
1-year	4.8	6.1	4.7	5.4	6.7	6.0	7.1	5.5	6.5	6.6
3-year	6.2	8.6	6.8	8.5	9.3	9.2	9.8	7.9	8.7	-
5-year	7.7	10.8	8.8	10.1	11.6	11.3	12.0	-	-	-

Hypertensive Heart Disease (I11, I13)										
7-day	0.7	1.0	1.0	0.9	1.3	0.8	1.1	1.1	1.2	1.0
30-day	1.7	2.6	3.0	2.5	3.3	2.4	2.6	2.2	2.6	2.5
1-year	5.7	7.9	7.6	6.4	7.5	6.4	6.0	5.5	6.2	5.5
3-year	10.1	11.4	12.0	10.4	12.1	9.4	9.2	8.3	8.5	-
5-year	11.7	13.4	14.0	12.6	14.7	11.9	10.7	-	-	-
Arrhythmia (I47-I49)										
7-day	0.4	0.6	0.6	0.5	0.5	0.5	0.6	0.6	0.5	0.7
30-day	0.8	1.2	1.0	1.0	1.0	1.0	1.1	1.0	1.0	1.3
1-year	2.0	2.6	2.7	2.4	2.6	2.4	2.6	2.3	2.3	2.6
3-year	3.6	4.3	4.2	4.2	4.4	3.8	3.9	4.0	3.8	-
5-year	4.9	5.6	5.5	5.7	5.7	5.0	4.9	-	-	-
Pulmonary Heart Disease (I26~I18)										
7-day	1.1	1.4	1.4	1.3	1.9	1.6	1.6	1.8	1.1	1.0
30-day	1.7	3.0	2.7	2.8	2.8	2.9	2.4	2.9	2.7	2.6
1-year	4.4	5.6	4.5	5.1	5.8	5.8	4.3	5.6	4.3	4.2
3-year	6.7	8.4	6.4	6.9	8.1	7.7	5.7	7.0	6.2	-
5-year	7.7	10.5	8.1	8.2	9.1	9.2	7.4	-	-	-
Ischemic Heart Disease (I20~I25)										
7-day	0.9	1.0	0.9	0.8	0.8	0.8	0.8	0.8	0.7	0.8
30-day	1.5	1.7	1.6	1.4	1.4	1.4	1.3	1.3	1.3	1.4
1-year	2.8	3.0	2.8	2.5	2.3	2.5	2.3	2.2	2.1	2.3
3-year	4.4	4.4	4.1	3.6	3.4	3.5	3.3	3.2	3.1	-
5-year	5.5	5.6	5.1	4.6	4.3	4.4	4.2	-	-	-

Table S11. Trends of case-fatality rates by subtypes of HD in Korean female, 2006 to 2015.

Variables	2006	2007	2008	2009	2010	2011	2012	2013	2014	2015
Heart Disease (I00-I09, I11, I13, I20~I51)										
7-day	1.3	1.5	1.5	1.6	1.6	1.7	1.6	1.7	1.6	1.8
30-day	2.6	2.9	2.7	2.8	2.8	2.9	2.9	2.8	2.8	3.1
1-year	5.1	5.4	4.7	4.8	4.8	4.9	4.5	4.6	4.6	5.0
3-year	7.7	7.6	7.0	7.0	7.0	6.9	6.5	6.7	6.5	-
5-year	9.6	9.5	8.8	8.6	8.5	8.6	8.1	-	-	-
Heart Failure (I50)										
7-day	1.8	2.7	3.2	3.5	3.1	3.3	3.6	3.3	3.5	3.5
30-day	4.1	6.1	6.4	6.7	6.8	7.1	7.3	6.8	7.1	7.1
1-year	11.6	14.1	13.5	13.6	13.5	13.7	13.2	13.1	13.7	13.7
3-year	18.1	20.5	19.6	19.8	19.7	19.8	19.2	19.3	19.6	-
5-year	22.2	25.2	23.9	23.9	23.6	23.6	23.2	-	-	-
Heart Disease Others (I00-I09, I30-I33, I39, I40-I46, I51)										
7-day	1.1	2.0	1.9	2.9	2.8	3.2	3.9	3.3	3.2	3.9
30-day	2.3	4.2	4.2	4.6	5.3	4.9	6.4	5.3	5.5	6.4
1-year	5.9	8.5	7.7	8.2	8.6	8.9	9.3	8.8	8.8	9.8
3-year	9.4	11.6	11.1	11.5	12.2	12.3	11.6	12.0	11.9	-
5-year	11.8	13.9	13.7	13.4	14.4	14.4	14.0	-	-	-
Valvular Heart Disease (I34-I38)										
7-day	1.0	1.3	1.0	1.1	1.5	1.5	1.9	2.1	1.9	2.2
30-day	2.0	3.1	3.4	2.9	3.2	4.0	4.4	4.5	4.8	4.4
1-year	5.0	7.5	7.6	6.9	7.3	8.4	8.9	7.8	9.0	8.6

3-year	9.2	10.5	12.3	10.3	10.8	11.6	13.2	12.1	12.7	-
5-year	11.4	13.4	14.8	12.7	13.3	14.6	16.3	-	-	-
Hypertensive Heart Disease (I11, I13)										
7-day	0.9	1.3	1.0	1.4	1.2	1.3	1.1	1.0	1.0	1.2
30-day	2.5	3.5	3.3	3.6	3.3	3.4	2.9	2.8	2.7	3.4
1-year	8.0	9.3	8.2	9.0	8.4	9.0	7.0	6.6	6.8	6.9
3-year	12.9	13.5	13.2	14.0	13.2	12.9	10.8	10.5	10.0	-
5-year	15.8	16.2	16.0	17.2	16.6	15.9	13.2	-	-	-
Arrhythmia (I47-I49)										
7-day	0.5	0.7	0.9	0.8	0.8	0.7	0.6	0.8	0.6	0.8
30-day	0.9	1.4	1.3	1.5	1.4	1.6	1.3	1.7	1.3	1.4
1-year	2.9	4.1	3.8	3.7	3.4	3.9	3.3	3.7	3.5	3.6
3-year	5.7	6.8	6.2	6.3	6.0	6.3	5.9	6.2	6.1	-
5-year	7.8	8.9	8.3	8.4	7.7	8.4	8.1	-	-	-
Pulmonary Heart Disease (I26~I18)										
7-day	1.8	1.8	2.4	1.6	2.1	2.4	2.8	2.0	2.2	1.6
30-day	3.2	3.3	3.5	3.1	4.1	3.9	4.1	3.5	3.5	2.9
1-year	5.4	6.7	6.4	6.4	7.0	6.6	7.3	6.6	5.8	5.4
3-year	7.6	9.7	9.0	9.4	9.7	9.4	9.5	9.3	7.9	-
5-year	9.5	12.5	10.7	10.7	11.9	11.3	10.9	-	-	-
Ischemic Heart Disease (I20~I25)										
7-day	1.2	1.5	1.3	1.3	1.4	1.4	1.3	1.3	1.3	1.3
30-day	2.1	2.5	2.2	2.2	2.2	2.2	2.1	2.0	2.1	2.3
1-year	3.9	4.5	3.8	3.8	3.7	3.6	3.4	3.4	3.4	3.6
3-year	5.7	6.1	5.5	5.5	5.2	5.0	4.8	4.9	4.7	-
5-year	7.1	7.4	6.8	6.7	6.4	6.1	5.9	-	-	-
